# Advancements in Aqueous Two-Phase Systems for Enzyme Extraction, Purification, and Biotransformation

**DOI:** 10.3390/molecules29163776

**Published:** 2024-08-09

**Authors:** Nikša Bekavac, Maja Benković, Tamara Jurina, Davor Valinger, Jasenka Gajdoš Kljusurić, Ana Jurinjak Tušek, Anita Šalić

**Affiliations:** 1 Faculty of Food Technology and Biotechnology, University of Zagreb, Pierottijeva 6, 10000 Zagreb, Croatia; nbekavac@pbf.hr (N.B.); maja.benkovic@pbf.unizg.hr (M.B.); tamara.jurina@pbf.unizg.hr (T.J.); davor.valinger@pbf.unizg.hr (D.V.); ana.tusek.jurinjak@pbf.unizg.hr (A.J.T.); 2 Faculty of Chemical Engineering and Technology, University of Zagreb, Marulićev trg 19, 10000 Zagreb, Croatia; asalic@fkit.unizg.hr

**Keywords:** aqueous two-phase system (ATPS), enzyme extraction, green solvents, liquid–liquid extraction (LLE), biotransformation

## Abstract

In recent years, the increasing need for energy conservation and environmental protection has driven industries to explore more efficient and sustainable processes. Liquid–liquid extraction (LLE) is a common method used in various sectors for separating components of liquid mixtures. However, the traditional use of toxic solvents poses significant health and environmental risks, prompting the shift toward green solvents. This review deals with the principles, applications, and advantages of aqueous two-phase systems (ATPS) as an alternative to conventional LLE. ATPS, which typically utilize water and nontoxic components, offer significant benefits such as high purity and single-step biomolecule extraction. This paper explores the thermodynamic principles of ATPS, factors influencing enzyme partitioning, and recent advancements in the field. Specific emphasis is placed on the use of ATPS for enzyme extraction, showcasing its potential in improving yields and purity while minimizing environmental impact. The review also highlights the role of ionic liquids and deep eutectic solvents in enhancing the efficiency of ATPS, making them viable for industrial applications. The discussion extends to the challenges of integrating ATPS into biotransformation processes, including enzyme stability and process optimization. Through comprehensive analysis, this paper aims to provide insights into the future prospects of ATPS in sustainable industrial practices and biotechnological applications.

## 1. Introduction

In recent years, there has been an increasing emphasis on conserving energy and environmental protection in various industries due to rising costs, decreasing energy resources, and ecotoxicological and hazardous effects on human health [1,2,3]. Addressing these issues in industrial processes necessitates exploring novel methods that consume less energy and focus on enhancing process energy efficiency. One such widely used energy-intensive process is liquid–liquid extraction (LLE), a technique extensively applied for separating components of liquid mixtures by using a solvent. LLE is instrumental in sectors such as pharmaceuticals, food processing, and industrial wastewater treatment [4]. Despite its wide application, traditional LLE often involves the use of organic solvents, which can be toxic and environmentally harmful [5]. This has led to a growing interest in developing greener alternatives. Green extraction technologies and green solvents are pivotal in advancing sustainable and eco-friendly processes in various industries, particularly in pharmaceuticals, cosmetics, and food [6,7]. Green extraction technologies emphasize the use of methods that reduce energy consumption, minimize waste, and utilize renewable resources. Techniques such as supercritical fluid extraction, microwave-assisted extraction, and ultrasonic-assisted extraction exemplify this approach, offering efficient, low-energy, and high-yield alternatives to traditional extraction methods [8,9]. Green solvents, including water, ethanol, supercritical CO_2_, and ionic liquids, replace conventional, often toxic, solvents [10,11,12]. These solvents are chosen for their biodegradability, low toxicity, and minimal environmental impact. Implementing these technologies not only aligns with environmental regulations but also enhances the sustainability profile of the final products, making them safer for consumers and the environment. As industries increasingly adopt green extraction technologies and solvents, they contribute to a circular economy, promote resource efficiency, and drive innovation in sustainable practices. A summary of the most important findings on the reduction of toxic solvent usage and the potential benefits for sustainable industrial practices in given in Table 1.

A promising approach that aligns with both efficiency and environmental goals is the aqueous two-phase system (ATPS). ATPS is a LLE technique that operates on the principle of phase separation using water-based polymer or polymer-salt systems, which can significantly reduce or eliminate the need for harmful organic solvents. This method has gained attention due to its compatibility with biological molecules and potential for environmental sustainability [17].

**Figure 1:**
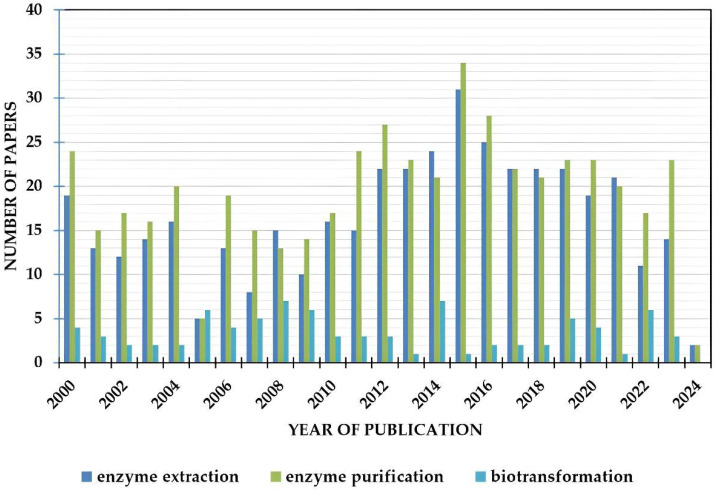
Number of publications according to Web of Science (WoS) in fields of ATPS for enzyme extraction, purification, and biotransformation.

ATPS can be highly effective for the extraction and purification of biomolecules, including enzymes, which are essential in numerous industries such as chemical, pharmaceutical, food, and biotechnology [18,19]. Enzymes are often targeted for their biological activity, and maintaining their functional integrity during extraction is crucial. Traditional methods for enzyme extraction can be cumbersome and may lead to the denaturation or loss of enzyme activity. ATPS offers a gentler and efficient alternative, preserving enzyme activity while providing high yields [20,21]. The importance of ATPS can be seen from the number of scientific papers published from 2000 to 2024 (Figure 1). The number of scientific papers on aqueous two-phase systems (ATPS) has seen significant growth from 2000 to 2024, reflecting the increasing interest and advancements in this field. This interest is driven by the versatility of ATPS in separating and purifying biomolecules, enzymes, and other biological materials, which is crucial for various biotechnological and pharmaceutical applications.

**Figure 2:**
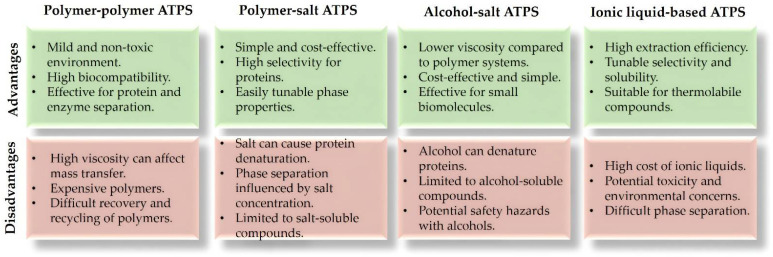
Overview of the most common types of ATPS, along with their advantages and disadvantages [26,27,28,29,30].

The advantages of ATPS over conventional methods include high purity of extracted products in a single step, scalability, and the use of water-based solvents, which are less harmful to the environment [22]. Commonly used systems involve at least two thermodynamically incompatible polymers, or polymer and salt or alcohol and salt [23]. Innovations in ATPS include the use of deep eutectic solvents and ionic liquids, which further enhance the stability and extraction efficiency of biomolecules [24,25]. The advantages and disadvantages of the most commonly used types of ATPS are presented in Figure 2.

**Figure 3:**
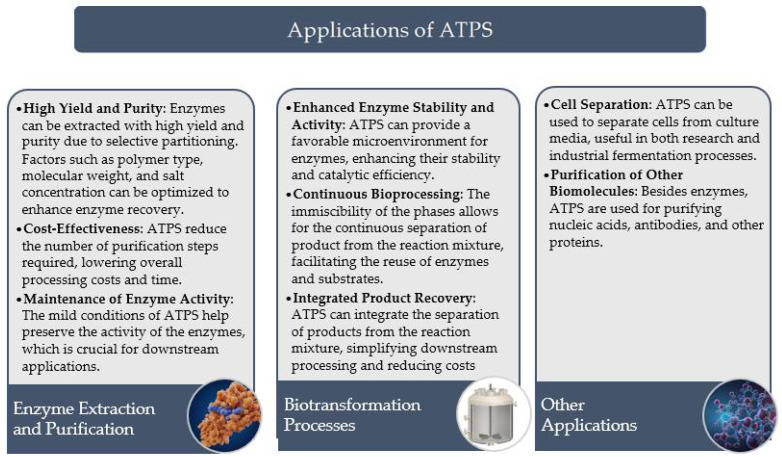
Application of ATPS.

This review paper aims to provide an in-depth overview of the principles, applications, and benefits of ATPS, particularly in the context of enzyme extraction and purification (Figure 3). It will also discuss the variables influencing the efficacy of ATPS, such as the choice of phase-forming components, system pH, and temperature, and explore the potential of ATPS in various biotransformations. Additionally, the paper will highlight recent advancements in ATPS technology and its implications for industrial and scientific applications.

## 2. Principles of Liquid–Liquid Enzyme Extraction

As previously mentioned, LLE is a mass transfer process achieved by bringing into direct contact two mutually immiscible or partially miscible liquids, between which the distribution of enzymes occurs [31]. The mass transfer from one phase to another happens by the laws of mass transfer and equilibrium distribution. Target molecules, like enzymes, are transferred from one liquid phase to another based on their partitioning behavior between the phases [32]. This partitioning is influenced by various factors that can be grouped in:

(i) Enzyme properties such as hydrophobicity, charge, molecular weight, bio-specific affinity, and conformation.
(ii) Phase partitioning system properties such as the polymer molecular weight and concentration, salt types and concentration, system pH, NaCl addition, temperature, and the number of cycles in partitioning [33].

According to Yousefi and Abbasi, the most important factors are the relative solubility of the enzyme in each phase, the composition of the solvent systems, and the operating conditions such as temperature and pressure [34]. Understanding the mechanisms underlying mass transfer and equilibrium distribution is essential for optimizing LLE processes in various applications, including the purification of bioactive compounds from natural sources, the separation of industrial chemicals, and the recovery of valuable products [35,36]. Furthermore, advancements in extraction equipment, optimization algorithms, and computational modeling techniques have contributed to the improvement of LLE efficiency and scalability [37].

Equilibrium in liquid–liquid systems (LLS) is significantly influenced by the chemical characteristics of dissolved substances and the solvents that form the LLE system [38]. The solvents must have certain desirable physical and chemical properties, among which are a great difference in densities between them and the starting solution of the solute mixture being separated, higher solubility of the solute in one of the phases, a large diffusion coefficient, and maximum selectivity of extraction for the desired substance in one of the phases [39,40]. The key principles and factors involved in LLE of enzymes are listed in Table 2. 

Additionally, the design and optimization of LLE systems involve considerations of thermodynamic properties, phase equilibria, and process parameters to achieve desired extraction yields and purity levels [41]. Because of the advances in mathematical modeling, optimization algorithms, and the development of novel experimental techniques, there has recently been better understanding and implementation of LLE processes across various industrial sectors, including pharmaceuticals and biotechnology [42,43].

## 3. Thermodynamics and Equilibrium of ATPS

ATPS are formed by mixing aqueous solutions of two mutually immiscible polymers, or polymers and salts [44]. When extracting compounds, it allows the formation of different phases without adding any organic solvents, increasing the green aspect of extraction [45]. One of these components must be a kosmotropic compound (isolating solvent), while the other is chaotropic. Kosmotropic compounds, characterized by smaller radii and higher charges, interact with water molecules and exert on the molecular organization of the water, therefore inducing the formation of two phases by attracting water molecules and displacing the solvent from the initial aqueous solution into the other phase. In contrast, chaotropic compounds, with larger radii and lower charges, exhibit weak interactions with water, disrupting its structural arrangement [46,47].

ATPS possess several properties that make them suitable for working with biological materials, including high water content in phases, although they are not miscible, low interfacial tension between phases, and, consequently, rapid establishment of equilibrium [48,49]. Since ATPS usually contain water in a proportion of 65–90% and the use of conventional, often toxic, organic solvents is not required, aqueous two-phase extraction is safe and environmentally friendly. Furthermore, this type of extraction is carried out under mild conditions, which, in addition to the aforementioned nontoxicity, means that the biomolecules retain their original conformation and activity [50]. Another important feature of ATPS is the possibility of easily modifying their properties by selecting different components and changing their concentrations, thus achieving considerable system flexibility as well as the desired selectivity and extraction efficiency [51]. The numerous advantages of ATPS make them one of the most promising green extraction technologies and have conditioned their application in an increasing number of processes. Some of these processes include the removal of pollutants from the environment and the isolation and purification of various biomolecules and cells from microorganisms and viruses, contributing to significant successes in current areas of biomolecules such as biocatalysis, gene therapy, antibody therapy, and the development of new vaccines [52,53].

**Figure 4:**
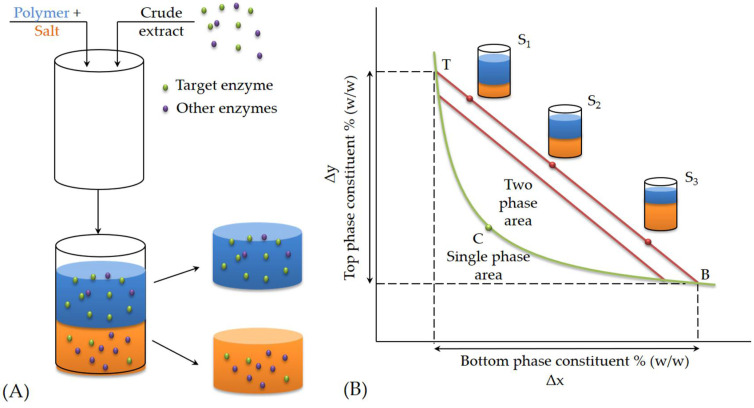
(**A**) Principle of phase separation and (**B**) phase diagram (TCB is binodal curve, C is critical point, TB is tie line, T and B are compositions of top and bottom phase).

The typical visual representation of an ATPS is a phase diagram. Such a system is actually tertiary, consisting of two polymers (or a salt and a polymer) and water, but for simplicity, it is often represented as binary. The curve in the phase diagram represents the binodal curve, which defines the heterogeneous region (two-phase system) from the homogeneous (one-phase system) [49]. In other words, if the system contains polymers (or a polymer and salt) in concentrations represented by a point above the binodal curve, two phases will form [54]. The obtained phases differ in composition: polymer 1 predominates in the upper phase, and polymer 2 (or salt) in the top phase (Figure 4A). Even with the construction of a phase diagram for potential ATPS systems, the thermodynamic stability of ATPS is not easy to predict.

As seen on the phase diagram, the increase in polymer and salt concentrations leads to the formation of two phases and the creation of ATPS, which leads to partial dehydration of the solutions (Figure 4B). The dehydration is partial because both phases remain water-rich, and the solutions are hydrated to a certain extent. Separation also occurs due to repulsive forces between anions and partially negative atoms in the polymer. Generally, interactions in ATPS occur through the hydration of oxygen atoms in the polymer or salt ions and interactions between salt cations and -O and =O groups in the polymer. The predominant interactions and the isolation effect depend on the prevailing interactions and whether cations predominantly interact with water or the polymer. If the charge density of the cation or the molar mass of the polymer is higher, their mutual interaction will be stronger [55].

The line connecting the points T and B is called the tie line. The tie line can be described by the equation of a line that passes through two points (T, B), and is determined by its slope (*k*) and length (*TLL*, tie line length). If the connecting line is described by the line equation (Equation (1)) [44]:

(1) y=k·x+l

then the *TLL* is given by the expression (Equation (2)) [44]:

(2) $$TLL=\sqrt{∆x^{2}+∆y^{2}}=\sqrt{{\left(x_{2}-x_{1}\right)}^{2}+{\left(y_{2}-y_{1}\right)}^{2}}$$

By arranging the Expression (2) and including the expression (Equation (3)) [44,49], which defines the slope of the tie line:

(3) $$k=\frac{y_{2}-y_{1}}{x_{2}-x_{1}}$$

it follows that:

(4) $$TLL=(x_{2}-x_{1})\cdot \sqrt{k^{2}+1}$$

The compositions of the phases at equilibrium are given by the intersections of the connecting lines with the binodal curve. Any two-phase system that can be represented by a point on the connecting line is always stratified into an extract and a raffinate of the same composition, but the volume ratio of the extract and raffinate phases is different, and depends on the ratio of the lengths of T and B.

A binodal curve can be described by a mathematical function, and by one that can reliably describe the equilibrium of a water two-phase system in a certain composition area. Thus, the equilibrium of the aqueous two-phase system, PEG_6000_-H_2_O-(NH_4_)_2_SO_4_, can be described by an exponential mathematical function of the form (Equation (5)) [49]:

(5) $$y=A\cdot e^{\left(-\left(C\cdot x+D\cdot x^{2}\right)\right)}$$

with parameters *A* = 0.664, *C* = −15.224, and *D* = 75.499. The function is valid for describing the thermodynamic equilibrium of the PEG_6000_-H_2_O-(NH_4_)_2_SO_4_ system at a temperature of 20 °C, i.e., in the range of connecting line lengths [0 < *TLL* < 51], where the coefficient of the connecting line direction is *k* = −2.21.

The thermodynamic equilibrium of ATPS can also be described by thermodynamic models such as NRTL and UNIQUAC. Thermodynamic models of aqueous two-phase systems describe changes in the composition of phases taking into account changes in temperature, concentration of components, molecular mass of polymers, distribution of one or more components between phases, pH, and ionic strength of the solution on the distribution of the extracted component. The distribution of substances between phases can be expressed through the distribution coefficient, *K* (Equation (6)) [49]:

(6) $$K=\frac{c_{E}}{c_{R}}$$

where $c_{E}$ is the equilibrium concentration of the substance in the extract and $c_{R}$ is the equilibrium concentration of the substance in the raffinate. When choosing an aqueous two-phase system, it is necessary to take care that the distribution coefficient is as small as possible (<<1) or as large as possible (>>1), but different from 1 [49].

In order to predict the behavior of ATPS, numerous models have been established, but most of them are empirical, since such systems are very complex and there is no comprehensive theory to describe them [56]. Since the partition coefficient is one of the most important variables for the design of the extraction process as well as for extraction with ATPS, different approaches are used to set up models that link the physico-chemical properties of the components to the partition coefficient [49]. The Albertsson model (Equation (7)) [44,49], which takes into account the influence of six different factors on the behavior of the system, and the partition coefficient are calculated as follows:

(7) $$lnK=lnK^{0}+lnK_{\mathrm{elec}}+lnK_{\mathrm{hfob}}+lnK_{\mathrm{afin}}+lnK_{\mathrm{siz}}+lnK_{\mathrm{conf}}$$

where *K*_elec_, *K*_hfob_, *K*_afin_, *K*_siz_, and *K*_sonf_ denote the contribution of the electrochemical factor, the hydrophobic factor, the biospecific factor, the size-dependent factor, and the conformation-dependent factor, respectively, while $K^{0}$ stands for other factors. The electrochemical factor refers to the separation caused by the electric potential, while the hydrophobic factor refers to the separation determined by the hydrophobic properties of the molecules. In addition, the biospecific affinity refers to the binding of certain molecules to certain sites on other molecules, the size refers to the size of the molecules themselves or their specific surface area, and the conformational factor refers to the conformations of the individual molecules [44]. The partitioning of biomolecule in ATPS is governed by thermodynamic principles that include changes in Gibbs free energy (Δ*G*), enthalpy (Δ*H*), and entropy (Δ*S*). Gibbs free energy indicates the spontaneity of the partitioning process. Negative Δ*G* means the process is spontaneous. Furthermore, enthalpy reflects the heat exchange during the partitioning. This indicates whether the process is exothermic (releases heat) or endothermic (absorbs heat). Finally, entropy represents the change in disorder. Positive Δ*S* suggests an increase in randomness [57,58].

Other factors may also be considered, such as the environmental factor, which depends on the type and concentration of salt, pH, temperature, and many other parameters, and depending on the components that make up the aqueous two-phase system and their properties, some of the above factors may dominate and determine the behavior of the system [44,49]. When using ATPS for enzyme extraction and/or purification, specific activity and purification factors are very important criteria of the process efficiency estimation. As presented by Brígida et al. [59], enzyme specific activity (*SA*) can be expressed as the ratio of enzyme activity (*EA*) and protein concentration ($c_{P}$) (Equation (8)), while the purification factor (*PF*) can be expressed as the ratio between specific activity of the enzyme after (*SA*) and before (*SA*_i_) the partitioning procedure (Equation (9)).

(8) $$SA=\frac{EA}{c_{P}}$$

(9) $$PF=\frac{SA}{{SA}_{i}}$$

**Figure 5:**
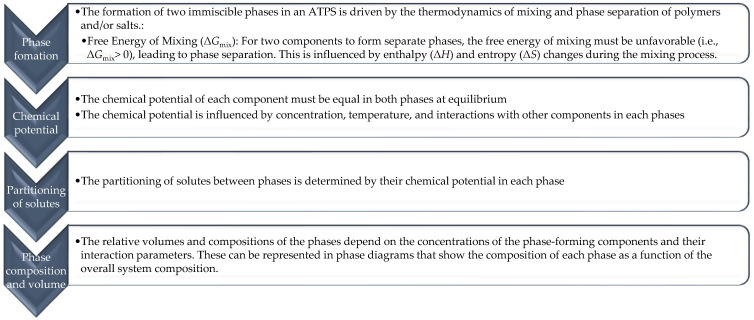
The principles of ATPS formation.

In general, the principles of ATPS formation of two immiscible aqueous phases and the partitioning behavior of solutes (like enzymes) between these phases can be described as presented in Figure 5.

ATPS can be formed by solving various polymers such as polyethylene glycol [60] and dextrane [61], but also deep eutectic solvents [62], ionic liquids [63], and organic [64] and inorganic salts [65] in water. According to Pereira and Coutinho, ATPS can be categorized as:

(i) Polymer/polymer ATPS that can be formed by mixing of (a) two nonionic polymers, (b) one nonionic and an ionic polymer, and (c) two charged polyelectrolytesnate (PSS);
(ii) Polymer/salt ATPS formed by the dissolution of a water-soluble polymer and inorganic (or organic) salt above critical concentrations;
(iii) Salt/salt ATPS;
(iv) Aqueous micellar two-phase systems (AMTPS);
(v) Ionic liquid-based ATPS with polymers, carbohydrates, amino acids;
(vi) Carbohydrate-based ATPS;
(vii) Copolymer-based ATPS;
(viii) ATPS composed of deep eutectic solvents;
(ix) ATPS composed of hydrophilic organic solvents [66].

Depending on the concentration and physical, chemical, and thermodynamic properties of these compounds, various combinations can lead to solvent separation into two phases. The partitioning of components in ATPS is governed by various driving forces that depend on the nature of the phase-forming components and the solutes being separated [67,68]. These driving forces include differences in chemical potential, hydrophobicity, electrostatic interactions, and molecular size and shape [68,69,70]. Overview of the driving forces for the partitioning in different types of ATPS is presented in Table 3:

The mutual solubility of solvents and the distribution of the enzyme between the two phases of a solvent system are defined by multiple interactions between solvents and the enzyme [82]. The most crucial role in enzyme transfer is played by the ability of molecules of one component from the mixture of dissolved substances to form hydrogen bonds with a component of the solvent or with a component of the dissolved mixture. Molecules with hydrogen atom donors act as hydrogen bond donors, while molecules or atoms in a molecule with free electron pairs act as hydrogen bond acceptors [83]. Some substances possess both properties. Accordingly, they are distinguished as follows:

(1) Substances possessing both donor and acceptor properties, such as molecules with hydroxyl groups (water, alcohols, phenols), amines, and carboxylic acids [84];
(2) Molecules possessing exclusively acceptor properties, such as ethers, ketones, aldehydes, and esters [85];
(3) Substances with donor molecules, such as chloroform, methyl chlorides, and ethylene chlorides [86];
(4) Substances that do not form hydrogen bonds, such as hydrocarbons, chloroform, ethylene chloride, and carbon tetrachloride [87].

Molecules possessing hydrophilic and hydrophobic parts have properties that depend on the ratio of these parts. Increasing the number of hydroxyl groups in a molecule increases its solubility in water [88]. When selecting a solvent for extraction, it is essential to ensure that the value of the distribution coefficient is as high as possible, that the solvent is chemically inert, i.e., does not react with the substances being extracted, and that it has a significant density difference from the starting phase. Preference is always given to solvents that are cheaper, less harmful, and less dangerous [89].

## 4. Enzyme Transfer in ATPS

The use of ATPS in enzyme extraction is studied in terms of phase-creating components in ATPS. To maximize enzyme partitioning and stability involves fine-tuning various factors such as pH, temperature, and the concentration of ATPS components. This optimization is crucial for enhancing the efficiency of enzyme separation and maintaining enzyme activity. The most important information about key factors affecting the enzyme separation in ATPS is given in Table 4.

In a study by Kaplanow et al., the mass transfer coefficients of lysozyme and bromelain were significantly lower compared to those of an organic compound in an organic/organic system, suggesting that the rapid equilibrium observed in ATPS is likely due to the substantial interfacial area created during mixing. By comparing the physicochemical properties of polymer/salt ATPS with other systems and analyzing the mass transfer coefficients of solutes with varying molecular weights, it is highlighted that molecular size plays a crucial role in influencing the mass transfer coefficient in these systems [76]. 

Ionic liquid-based ATPS are used for purifying lipolytic enzymes, specifically lipases, which play a crucial role in various industries due to their chemo-selectivity and stereo-selectivity [98]. By comparing the performance of ionic liquid-based ATPS with conventional PEG-based systems, it has shown the superior purification efficiency of IL-based ATPS for enzymes, showcasing the potential of ionic liquids as novel separation agents for extracting macromolecules like proteins and enzymes with high activity, stability [99], and extraction efficiency. The use of ionic liquids (ILs) and inorganic salts in ATPS for the separation and purification of enzymes shows higher recovery rates and improved process properties compared to conventional methods [100]. Some examples of using ATPS in enzyme separation and purification are given in Table 5.

Specifically, the integration of hydrophilic ionic liquids in enzyme separation processes is highlighted as an efficient and cost-effective approach since enzyme activity can be preserved and the general costs of extraction are decreased [120]. Enzyme partition and purification on IL-based ATPS involve using specific ionic liquids (ILs) in combination with phosphate buffer solutions to purify enzymes. The choice of ILs impacts the purification process, with factors such as alkyl chain length, cation core, and anion moiety influencing the purification efficiency [121]. Varying the alkyl chain length of ILs, isoelectric point, hydrophobic/hydrophilic nature, and the type of ions affects the purification parameters, such as purification factor and partition coefficient, highlighting the importance of IL properties in enzyme purification processes [122]. It was shown that ionic liquid-based ATPS offers superior purification performance, with higher purification factors and enzyme recovery efficiencies compared to polymer-based systems, demonstrating the potential of ionic liquids in enzyme purification processes [123]. Ionic liquid-based aqueous two-phase systems were effectively utilized for purifying lipase from a bacterial fermentation broth, showcasing high purification factors and enzyme recovery efficiencies. The results demonstrated the superior efficiency of the IL-based ATPS compared to traditional polymer-based systems, highlighting its potential for enhancing extraction capabilities in bioseparation processes [124]. Further exploration into applying this purification technique to other enzymes or proteins is warranted to fully assess its versatility and effectiveness in diverse biotechnological applications [125].

The use of hydroxyl ammonium-based ionic liquids for protein extraction in an ATPS offers advantages such as wide liquid ranges, low volatilities, and good thermal stability, making them suitable for extracting proteins without denaturation [126]. Their use shows promise for providing a biocompatible environment for the extraction and purification of proteins, offering a potential alternative to traditional protein purification methods [127].

Liquid–liquid equilibrium data for ATPS with PEG 1500, sodium citrate/citric acid, and water were obtained at different pHs and temperatures, showing that pH influenced the biphasic region. α-amylase partitioning was studied concerning pH, temperature, and tie line length (TLL), revealing that the enzyme’s behavior varied with pH and temperature changes. Isothermal titration calorimetry (ITC) assays were conducted to understand the intermolecular interactions involved in α-amylase partition, showing that the process was entropically driven and accompanied by endothermic heat at specific pH and temperature conditions. The yield parameter (95.373%) indicated the practicality of using ATPS for α-amylase purification, showcasing the potential applicability of the studied systems in enzyme separation processes [128].

The optimization of the purification of serine protease from mango peel using a polyethylene glycol (PEG)/dextran-based aqueous two-phase system (ATPS) was conducted by investigating the impact of various parameters such as PEG molecular weight, tie line length (TLL), NaCl concentration, and pH on the partitioning, purification factor, and yield of serine protease in the PEG/dextran ATPS using response surface methodology (RSM). There was a significant effect of PEG selection on the efficiency of serine protease purification, with the most substantial impact observed on the purification process. The addition of 4.5% NaCl to the system led to a significant increase in the partition coefficient, possibly due to the higher hydrophobicity of serine protease compared to other protein contaminants. NaCl alters the interaction between hydrophilic polymers and enzymes, modifying the partitioning behavior in the ATPS, followed by increased yield. The hydrophobic interaction between PEG and the hydrophobic surface of serine protease was significantly increased. However, higher salt concentrations can have a negative effect on enzyme partitioning due to the unequal distribution of salt between the phases, affecting the chemical potential of the solute [129]. The optimized conditions achieved a high partition coefficient (84.2), purification factor (14.37), and yield (97.3%) of serine protease, demonstrating the feasibility of purifying the enzyme with high efficiency and yield using PEG/dextran ATPS [130].

ATPSs composed of pH-responsive polymers PADB4.91 and PADB4.06 were successfully established for the separation of transglutaminase, with high polymer recovery rates exceeding 96%. The partitioning of crude transglutaminase in the formed ATPS was investigated, with optimization studies on various parameters such as crude enzyme load, pH (ranging from 6.50 to 7.80), polymer concentrations, and types and concentrations of salts. In the presence of 60 mmol/L MgSO_4_ and at pH 7.00, a 3% PADB4 and 91/2% of PADB4.06 ATPS achieved an enzyme recovery of 96.51%, a partition coefficient of 4.23, and a purification factor of 3.73 for transglutaminase. The zeta potential measurements of the polymers at different pH values indicated that adjusting the pH to the isoelectric points allowed for the precipitation of the polymers, enabling their recycling [131]. The Ls54/Dextrin ATPS was shown to be a promising method for cutinase recovery, offering approximately 65% enzyme recovery with a purification factor of 6.92 at optimal conditions of 22% (*w*/*w*) Ls54 and 12.5% (*w*/*w*) Dextrin at pH 8.0 and 295.15 K. The temperature is especially shown to be a significant influence for the formation of the ATPS in general [132]. 

In Ls54/Dextrin ATPS, temperatures above 304.15 K caused turbidity and phase separation, while changes in pH from 6 to 9 do not significantly affect the phase volume ratio in the system. The enzyme recovery shows the potential of using low-cost starch derivatives such as Dx in ATPS, offering economic advantages over traditional systems using expensive polymers such as dextran [133].

L-asparaginase is extensively utilized for remission in acute lymphoblastic leukemia and other malignant neoplasms, driving the search for novel enzyme sources and efficient purification methods. Application of an aqueous two-phase micellar system (ATPMS) using Triton X-114 for the purification of fungal L-asparaginase produced by *Penicillium* sp.–encoded 2DSST1 was isolated. By optimizing the extraction, a maximal enzyme activity of 2.33 IU/mL was achieved. The ATPMS extraction gave a promising purification factor of 1.4 and 100% yield, highlighting its cost-effectiveness and potential to reduce industrial production costs through non-chromatographic techniques [134]. Similarly, the study on *Bacillus* sp. 11/3 inulinase used ATPS, varying the concentrations of polyethylene glycol (PEG) and salts to develop phase diagrams and achieve optimal purification. The selected ATPS system with 26% PEG1000 and 26% MgSO_4_ yielded a purification factor of 4.65, demonstrating the system’s effectiveness in enzyme purification [135].

Another innovative approach for protein extraction uses a deep eutectic solvent (DES)-based aqueous two-phase system. DES-based ATPS can be classified as ternary, quaternary, or pseudo-ternary systems depending on the specific components involved and their interactions [136]. For example, Passos et al. [136] reported that ABTS composted of carboxylic-acid-based DES are quaternary systems, while Farias et al. [137] developed pseudo-ternary ABTS based on the DES composed of choline chloride and sugars. By comparing several choline chloride (ChCl)-based DESs, DES ChCl-glycerol was found to be the most effective solvent for protein extraction due to its efficiency and lower impact on protein structure [138]. The experiments determined optimal conditions for the extraction process, including the amount of DES, salt concentration, protein mass, shaking time, temperature, and pH, which maximized protein recovery while maintaining protein integrity. UV-Vis, FT-IR, and circular dichroism (CD) spectra analyses confirmed that the protein’s conformation remained unchanged during the extraction, indicating that the DES-ATPS method is gentle and preserves the biological functionality of the proteins [139]. DES-ATPS showed high extraction efficiency with a slightly reduced selectivity when multiple proteins were present, suggesting room for improvement in specificity toward particular proteins [62]. Similarly, Xu et al. [140] showed that activity and conformation of the lysozyme was well kept in ATPS composed of DES (tetrabutylammonium bromide:glycolic acid) and Na_2_SO_4_. The author presented that more than 98% of lysozyme was transferred into the DES-rich phase at the optimum condition and that the activity of lysozyme after the process of extraction retained 91.73% of initial activity. Furthermore, Cai et al. [141] developed ATPS based on a pH-responsive polymeric DES and phosphate salt. This novel PDES-based ATPS was used to extract aromatic amino acids. The extraction efficiencies for tyrosine, phenylalanine, and tryptophan reached 95.25%, 99.05%, and 99.10%, respectively. It is very important to emphasize that by adjusting pH, PDES was recycled and reused. Zhuang et al. [142] and Pereira et al. [143] used DES-based ATPSs for extraction of phycocyanin an intracellular protein produced in *Spirulina* sp. The first group of authors used ChCl-Urea/K_2_HPO_4_ system and achieved extraction efficiency of 94.2% and yield of 92.0%. The second group of authors used natural deep eutectic solvents in ATPSs and achieved extraction efficiency of 99% and partitioning coefficient of 29.4%. These findings indicate that the DES-ATPS approach is a promising green alternative for protein extraction, offering high efficiency and environmental benefits over traditional methods. The study lays the groundwork for further optimization and application of this technology in bioseparation processes.

Ginsenoside CK is recognized for its pharmacological activities but is challenging to prepare effectively. An integrated production and extraction method was developed using DES-ATPS to enhance the bioavailability of ginsenoside CK through enzymatic hydrolysis. Ginsenoside CK was extracted using choline chloride-based DES in combination with K_2_HPO_4_ [144].

The recovery and reuse of phase-forming components in aqueous two-phase systems (ATPS) are crucial for sustainability and economic efficiency [145]. Efficient recovery methods reduce waste and operational costs by enabling the reuse of polymers, salts, and other agents [146]. Techniques like ultrafiltration, precipitation, and evaporation are commonly used, with polymers such as polyethylene glycol being precipitated and re-dissolved and salts recovered through crystallization [147,148,149,150,151]. Closed-loop systems and advanced methods like membrane technologies and selective extraction enhance recovery efficiency and component integrity. This practice reduces the environmental footprint and supports the large-scale industrial application of ATPS, aligning with green chemistry and sustainable development goals.

## 5. Biotransformations 

The increasing demand for biocatalytic processes as alternatives to traditional chemical routes for the synthesis of compounds highlights challenges such as limited enzyme stability and low reaction rates due to restricted process parameters [152]. The changes in water activity and hydration levels within the ATPS affect the enzyme’s catalytic activity, so the optimization of the ATPS can be conducted properly [153]. Some examples of using ATS for biotransformations are given in Table 6.

Despite the advantages of ATPS, there are challenges that need to be addressed for widespread adoption in biomanufacturing industries, such as understanding the maximum capacity of these systems, predictive design limitations, and comparison with existing platforms in terms of economic and environmental sustainability. The list of main challenges working with ATPS along with proposed solutions is given in Table 7.

Experimental design methodologies have been used to optimize the purification process conditions in ATPS, but detailed models predicting the partition behavior of biomolecules are currently lacking. The use of thermoseparating polymer-based aqueous two-phase systems (ATPS) was researched for the enzymatic hydrolysis of starch. Different ATPS, specifically those with polymers such as PEO–PPO-2500 and salts such as ammonium sulfate and magnesium sulfate, influence the partitioning and activity of enzymes involved in starch conversion, namely α-amylase and amyloglucosidase.

Various ATPS were tested for their ability to partition α-amylase and amyloglucosidase. The study found that enzyme partitioning is heavily influenced by the concentrations of the phase-forming components. Notably, the PEO–PPO-2500/MgSO_4_ system demonstrated significant promise for starch hydrolysis applications due to favorable partitioning behaviors. The use of ATPS led to improved starch hydrolysis compared to conventional methods, with the PEO–PPO-2500/MgSO_4_ system showing a notable increase in the yield of maltose and glucose, which suggests enhanced enzymatic activity and faster hydrolysis rates. The advantages of thermoseparating ATPS include the ability to recycle the polymer phase, reducing operational costs and environmental impact. The polymer’s phase separation can be controlled by adjusting temperature, making it a versatile tool for biotechnological applications; hence, thermoseparating ATPS can significantly enhance the efficiency of enzymatic starch hydrolysis, positioning it as a viable method for industrial applications where starch conversion is required [167].

There was development and application of a pH-responsive aqueous two-phase system (pH-ATPS) utilizing sodium citrate and a recyclable pH-responsive polymer, PADB6.8. This system was designed to optimize the bioconversion of cefprozil, a semi-synthetic cephalosporin antibiotic, by improving yield and reducing product hydrolysis through effective phase separation. The pH-responsive polymer PADB6.8 features the ability to undergo phase transitions in response to pH changes, aiding in the extraction process by enabling easy recovery and recycling of the polymer. The system enabled effective separation of the bioconversion product into the polymer-rich phase, reducing product inhibition and hydrolysis. This setup improved the yield of cefprozil significantly compared to traditional methods. The use of pH-ATPS offers a biocompatible, low-cost, and environmentally friendly alternative to traditional solvent-based extraction methods. The system’s low interfacial tension and the recyclable nature of the polymer contribute to its sustainability [168].

ATPSs’ potential for the synthesis and recovery of cyclodextrins (CDs) using *Bacillus cereus* cyclodextrin glycosyltransferase (CGTase) was conducted. The optimal conditions for the extractive bioconversion were achieved using an ATPS composed of 7.7% (*w*/*w*) polyethylene glycol (PEG) 20,000 and 10.3% (*w*/*w*) dextran T500, with a volume ratio of 4.0. This setup allowed for effective enzymatic conversion of starch and subsequent transfer of CDs to the top phase. These results indicate that the ATPS is an effective and sustainable method for the bioconversion and recovery of cyclodextrins, offering significant advantages over traditional methods in terms of process efficiency and environmental impact [169]. Another study done by Lin et al. (2016) showed the ability to produce gamma-cyclodextrin (*γ*-CD) using *Bacillus cereus* cyclodextrin glycosyltransferase (CGTase) in a polymer-salt ATPS. The research focused on optimizing the bioconversion and purification of *γ*-CD into a single-step process using ATPS composed of 30% (*w*/*w*) PEG 3000 and 7% (*w*/*w*) potassium phosphate. Under these conditions, a concentration of 1.60 mg/mL of *γ*-CD was recovered after 1 h of bioconversion, indicating efficient production and separation efficiency. The *γ*-CD predominantly partitioned to the top phase with more than 81.88% recovery, while CGTase mainly stayed in the salt-rich bottom phase, facilitating its reuse. The system supported successful repetitive batch processes, indicating the potential for scalable operations [170].

Extractive bioconversion of poly-β-caprolactone with lipase in ATPS was optimized. A pH value of 7.0, temperature of 40 °C, 19% (*w*/*w*) PEG 3000, and 8.1% (*w*/*w*) potassium phosphate for forming an APTS is required to obtain 79.8% of the products (monomeric and dimeric forms of β-caprolactone) in the upper phase, and 42.0% of the lipase was effectively partitioned into the lower phase. Temperature, volume ratio, and presence of NaCl were significant variables affecting the partitioning efficiency of hydrolyzed PCL and lipase. Qualitative analysis using methods such as GC-MS/MS, DSC, and GPC revealed detailed information about the molecular structure and thermal properties of the products, confirming the effectiveness of the ATPS in preserving the enzymatic activity and enhancing the bioconversion process [171].

Optimizing the enzymatic hydrolysis of xylan into xylobiose and xylotriose using ATPS utilized PEG and sodium citrate to enhance the separation and yield of xylo-oligosaccharides, which are beneficial for stimulating the growth of intestinal bifidobacteria. The ATPS significantly increased the concentrations of xylobiose and xylotriose compared to traditional aqueous systems. Specifically, the ATPS yielded xylobiose and xylotriose concentrations of 2.12 g/L and 1.32 g/L, respectively, which were notably higher than those obtained from the aqueous system (1.08 g/L xylobiose and 0.52 g/L xylotriose). The xylanase partitioning within the ATPS was done by maintaining enzyme stability and achieving efficient substrate conversion. This optimized partitioning was critical for maximizing product yield while minimizing enzyme loss [172].

ATPS were also explored for the enzymatic conversion of sugarcane bagasse into sugars. They can effectively partition enzyme inhibitors (like sugars), thereby potentially increasing the hydrolysis efficiency by minimizing product inhibition. Overall, the potential of ATPS in improving the enzymatic conversion of lignocellulosic materials by reducing enzyme inhibition and optimizing reaction conditions is highlighted [173]. It is shown that the ATPS approach to biotransformations not only enhances the efficiency of production but also aligns with sustainable practices by minimizing waste and reusing the biocatalyst, demonstrating a significant advancement in bioprocessing technologies for industrial applications. These systems could potentially be adapted for other bioconversion processes, promoting greener and more efficient industrial practices.

## 6. Potential

ATPS offer significant potential for enzyme extraction and biotransformation due to their unique advantages, such as mild operating conditions, selective partitioning, scalability, cost efficiency, and environmental friendliness. These systems enable the extraction of enzymes with high yields by selectively partitioning them into specific aqueous phases, achieving high purity with minimal impurities, and maintaining enzyme activity under mild conditions. ATPS are versatile and can be adapted to different enzymes and extraction requirements by adjusting the type and concentration of phase-forming components. This flexibility is crucial for the efficient separation of enzymes used in various applications, from industrial processes to pharmaceuticals. Due to their advantages, there are examples of successful industrial applications of ATPS (Table 8).

These systems are promising for the recovery of high-value bioproducts due to their ability to operate under mild conditions that preserve the activity of sensitive molecules [24]. Moreover, ATPS with ionic liquids and deep eutectic solvents has shown potential for expanding the range of bioactive compounds that can be recovered, thus enhancing the utility of ATPS in industrial applications [178]. In biotransformation, ATPS provides a favorable microenvironment that increases enzyme stability and activity, facilitates continuous biotransformation processes, and integrates separation with reaction steps. This integration helps to reduce inhibitory effects and side reactions, improve reaction yields, and simplify downstream processing. For example, ATPS can be used in the pharmaceutical industry for the extraction and stabilization of enzymes for drug synthesis, in the food industry for the processing of enzymes such as amylases and proteases, and in the biofuel sector for the continuous production of bioethanol and biodiesel. In addition, ATPS are of central importance in environmental biotechnology for enzyme extraction in bioremediation and waste treatment. Despite the challenges of optimization and scale-up, the future prospects of ATPS are promising. Ongoing research is focusing on sustainability, cost reduction, and integration with other technologies to strengthen its role in biotechnological applications. Future perspectives include the use of ATPS in conjunction with molecular modeling to optimize conditions and expand their application in biotechnology [179].

**Figure 6:**
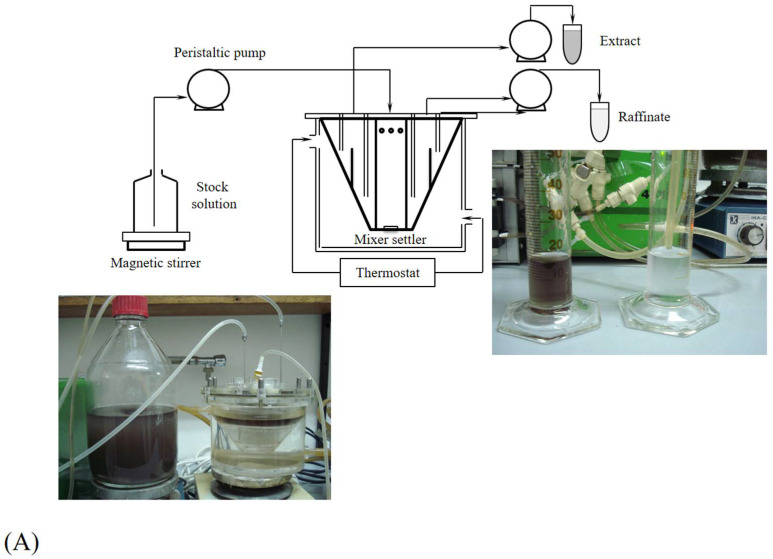

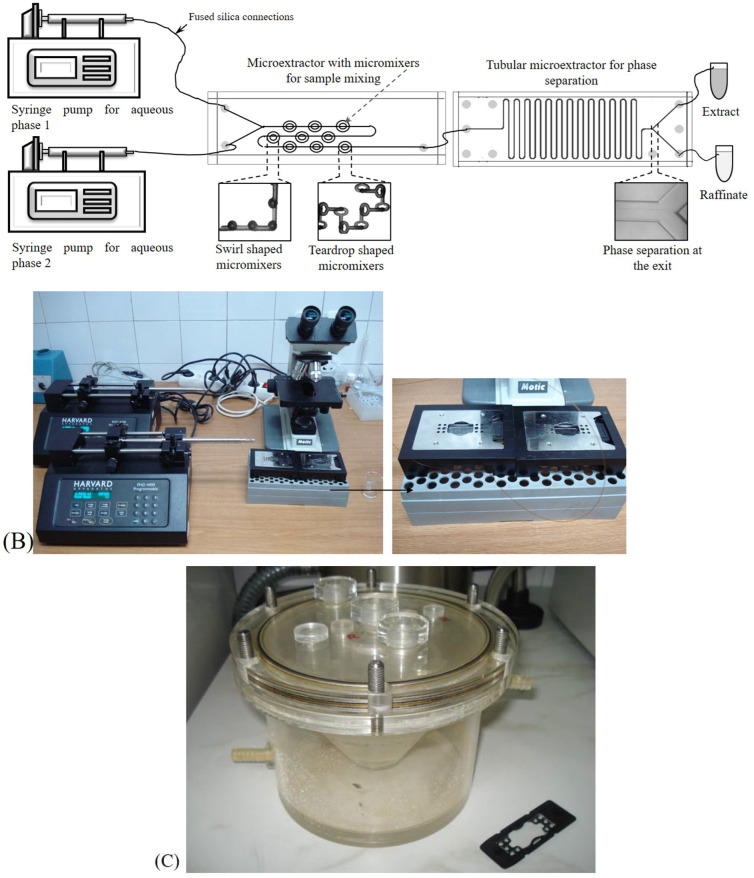
Examples of using ATPS in (**A**) macroextraction system, (**B**) microextraction system, and (**C**) comparison in size of macro-and microextractor.

ATPS can be effectively combined with both macro and micro extractors (Figure 6), enhancing the efficiency and scalability of enzyme extraction and biotransformation processes. By integrating ATPS with macro extractors such as continuous flow systems, centrifugal extractors, and agitated tank extractors, the process benefits from rapid phase separation, increased throughput, and the ability to handle large volumes, making it ideal for industrial-scale applications. As presented by Šalić et al. [180], ATPS based on natural deep eutectic solvents coupled with microfluidic devices ensure 98.50% extraction efficiency of lipase for a residence time of 30 s, which represents a significant improvement compared to batch processes for a similar extraction efficiency (94.70%) process lasting for 30 min. This combination allows for continuous, uninterrupted processing, thereby increasing productivity and cost-effectiveness while preserving enzyme activity under gentle conditions. On a smaller scale, the integration of ATPS with microextractors, including microfluidic devices, microscale centrifuges, and microchannel extractors, enables precise control over the extraction process. These setups facilitate efficient separation of enzymes with high selectivity, minimal reagent usage, and rapid phase separation, which is particularly beneficial for research, high-value applications, and analytical purposes. For example, Božinović et al. [181] investigated the application of ATPS systems based on polyethylene glycol (PEG1540) and various salts (sodium sulfate, sodium citrate dihydrate, sodium formate, sodium potassium tartrate tetrahydrate, and ammonium sulfate) for continuous microfluidic extraction of xylanase. Based on their results, ATPS sodium citrate dihydrate-H_2_O-PEG1540 proved to be the most effective system for xylanase purification by extraction, and using a microfluidic device, the extraction efficiency of *E* = 99.59% and purification factor of 6.61 ± 0.07) were obtained in 1.03 min, indicating the proposed process is effective for the purification of xylanase.

The fusion of ATPS with both macro and micro extractors not only improves the yield and purity of extracted enzymes but also enhances process flexibility, scalability, and environmental sustainability by reducing the reliance on organic solvents. For example, polymer/salt ATPS can efficiently be used for removing methyl orange dye in a microfluidic system. This combined approach leverages the advantages of both systems, offering a versatile and powerful solution for diverse applications in biotechnology and industrial processes [182]. As described by Ahmed et al. (2021) [183], quantifying protein–protein binding, probing for conformational changes, or monitoring enzyme activity have been performed with ATPS. According to Flora et al., in 2023 [184], microfluidics and lab-on-chip systems could potentially revolutionize medical diagnostics. The same scientists examined the identification of prostate-specific antigen using an immunoassay carried out in a microfluidic device based on microbeads that had been collected and purified from a serum sample using an aqueous biphasic system. They showed that ATPS composed of tetrabutylammonium chloride or tetrabutylphosphonium bromide and polyethylene glycol 1000 ensured a high extraction yield of PSA; therefore, the platform designed for PSA detection was able to achieve limits of detection of about 5 ng/mL. This point-of-care system demonstrated efficacy in obtaining dependable findings within the pertinent range for PSA diagnosis, given the PSA clinical range of 4–10 ng/mL.

The study by Oliva et al. [185] found that ATPS can significantly modulate enzymatic activity. In ATPS, the enzyme activity was altered, showing a marked decrease in turnover number kcat from 0.93 s⁻^1^ to 0.33 s⁻^1^ at ambient pressure, while the Michaelis constant (*K_M_*) remained roughly constant. High hydrostatic pressure (HHP) increased both *K_M_* and kcat in a buffer solution, indicating that pressure decreases enzyme–substrate affinity but accelerates the turnover number. However, in ATPS, pressure had no significant effect on these kinetic constants, suggesting that ATPS can counterbalance the effects of pressure on enzyme activity. Furthermore, ATPS can also be efficiently used for the formation of droplets in flow-focusing microchannels, taking into account that an essential first step in many biomedical and bioengineering applications is biosample encapsulation [186,187].

The combination of ATPS with membrane chromatography enhances the downstream processing efficiency of bioactive peptides. ATPS extraction, when coupled with membrane chromatography, could achieve a total product recovery rate of 99.9% for monoclonal antibody purification. The study explored the binding capacity of membranes in protein A, ion exchange, and hydrophobic exchange membrane chromatography under different conditions of polyethylene glycol (PEG), salt, and protein concentrations [188]. Such methods could potentially be used for the purification of highly valuable enzymes. The paper by Aguilar et al. [189] compares two processes for the purification of penicillin acylase (PA) from *E. coli*: traditional ion-exchange chromatography and ATPS, which were found superior in terms of enzyme recovery and economic efficiency. ATPS demonstrated a 97% recovery of PA with a purity factor of 3.5, compared to the chromatography process, which had a 48% recovery and a purity factor of 5.7. Economically, ATPS reduced the gross cost of separation by 37%, showcasing a substantial decrease in the number of unit operations required—from seven to four—thus simplifying the process and reducing costs. These findings advocate for the substitution of traditional chromatography methods with ATPS in the purification of PA, offering both higher efficiency and cost-effectiveness. Furthermore, Sharma et al. [103] used a 3D-baffle micromixer combined with a phase separator meticulously designed for automated selective separation of laccase. The author presented 88% extraction efficiency of laccase using APTS in a microfluidic system compared to 16.3% efficiency in the batch system.

The study by Vobecká et al. [190] explores the continuous-flow synthesis of cephalexin in a microfluidic device using ATPS: cephalexin can be effectively synthesized in a kinetic regime using a microfluidic setup, which allows for the in situ extraction of the antibiotic and recycling of the enzyme penicillin acylase. The system operates efficiently for at least five hours and incorporates a microdialysis unit to remove the side product, phenylglycine, thus preventing system clogging. The optimized ATPS composition included phosphates, polyethylene glycol, and water, which facilitated high enzyme reuse and product separation efficiency. This continuous synthesis method offers a promising alternative to traditional batch processes by improving the reaction yield, reducing reaction time, and enabling easier downstream processing due to the effective separation of the enzyme and reaction product. Meng et al. [191] also presented an innovative approach for enhancing enzymatic reactions using a microfluidic device with ATPS featuring parallel-laminar flow. This method significantly accelerates enzymatic reactions, specifically using urease, by combining reaction and separation processes into a single step. Key results include a 500-fold increase in reaction rate compared to conventional ATPS in a beaker setup and effective product separation due to differential phase partitioning. The ATPS in the microfluidic device optimized factors such as flow rates, substrate concentration, and residence time to enhance reaction efficiency and product recovery. This study provides a promising technology for high-efficiency biotransformations with practical applications in the biomedical, pharmaceutical, and food industries. Similarly, Jakob et al. [192] optimized the apparatus for continuous aqueous two-phase flotation in co-current or counter-current flow and showed that co-current mode increased the separation efficiency of biomolecules by about 14%.

Based on the before-mentioned, strengths, weaknesses, opportunities, and threats using ATPS systems for enzyme extraction, purification and biotransformation can be recognized as follows:

(i) Strengths: selectivity and efficiency, mild operating conditions, scalability, eco-friendly, cost-effectiveness;
(ii) Weakness: optimization requirements, challenging phase components recovery, limited solvent options, phase separation time;
(iii) Opportunities: Integration with other technologies, applications of ATPS in biotransformations, development of novel phase-forming systems like deep eutectic solvents, tailoring of ATPS for specific industrial application;
(iv) Threats: competition with other purification technologies, economic viability, and disposal of large volumes of phase-forming components still represents environmental concern.

## 7. Conclusions

The studies on ATPS have extensively shown their utility for the separation and purification of enzymes due to their ability to partition them based on surface properties influenced by the ATPS composition by using the differences in their surface properties, such as hydrophobicity and charge. These systems can be customized by altering the phase components, such as polymers and salts, to enhance selectivity for specific enzymes and favor their reactions. It is indicated that ATPS not only can preserve but can also enhance enzymatic activity during processes, making them particularly valuable for sensitive biotransformations where maintaining protein function is critical. The integration of ATPS with other separation techniques, such as chromatography or additional phase modification strategies, can further refine purity and yield, making this approach highly versatile for industrial applications. Future developments may focus on optimizing ATPS compositions using computational models and novel materials like ionic liquids to enhance performance and expand the range of applications, including the recovery of more complex biological entities like virus-like particles and high-value bioproducts. The ongoing research and development in ATPS is set to refine their capabilities and expand their application in biotechnology and beyond, driven by their efficiency, adaptability, and compatibility with green chemistry principles.

## Figures and Tables

**Table 1:** Summary of the most important findings on the reduction of toxic solvent usage and potential benefits for sustainable industrial practices [13,14,15,16].

Aspect	Findings
Reduction technique	Use of green solvents (like water and ethanol) instead of toxic solvents. Implementation of minimal solvent or solvent-free processes. Development of solvent recycling and recovery systems. Adoption of alternative technologies like supercritical CO_2_, ionic liquids, and deep eutectic solvents.
Environmental benefits	Decreased air, water, and soil pollution. Lower greenhouse gas emissions. Reduced chemical waste generation and disposal issues.
Health and safety benefits	Improved worker safety due to reduced exposure to toxic chemicals. Enhanced workplace conditions and reduced need for protective equipment.
Economic benefits	Cost savings from reduced need for solvent purchase and disposal. Potential for new market opportunities in green products.
Industrial applications	Chemical manufacturing using more sustainable reaction mediums.

**Table 2:** Key principles and factors involved in liquid–liquid enzyme extraction.

Key Principle	Key Factor
Partition Coefficient	*Hydrophobicity/Hydrophilicity:* Enzymes with hydrophobic regions may preferentially partition into a phase containing a higher concentration of hydrophobic components (e.g., organic solvents or polymers). *Charge:* Enzymes with charged amino acid residues can interact with ions in the aqueous phase, influencing their partitioning behavior. *Molecular Size and Shape:* Larger enzymes or those with specific structural features may exhibit different partitioning behavior due to steric hindrance or interactions with phase components.
Phase Composition	*Type and Concentration of Solutes:* Salts, polymers, or organic solvents in the phase influence the solvation environment and interactions with enzymes. *pH and Ionic Strength:* pH affects enzyme stability and ionization state, which in turn affects partitioning behavior.
Operating Conditions	*Temperature:* Extraction temperature can influence enzyme stability and phase equilibrium. Generally, milder temperatures are preferred to maintain enzyme activity. *Agitation and Mixing:* Ensures effective contact between the two phases, promoting mass transfer and equilibration of enzyme distribution. *Extraction Time:* The duration of extraction affects the extent of partitioning and should be optimized to achieve maximum yield and purity.
Selectivity and Specificity	*Selective Solubility:* Different enzymes have varying affinities for organic solvents, polymers, or aqueous phases based on their structural and chemical properties. *Affinity Ligands:* In some cases, ligands or affinity agents specific to the enzyme can be incorporated into one phase to enhance selectivity.
Recovery and Purification	*Separation Techniques:* After extraction, methods such as phase separation, centrifugation, or filtration are employed to separate the phases and recover the enzyme. *Purity Enhancement:* Multiple extraction cycles or additional purification steps may be required to achieve the desired level of purity for specific applications.

**Table 3:** The driving forces for the partitioning in different types of ATPS.

Type of ATPS	Driving Forces	Reference
Polymer–polymer	*Hydrophobic interactions:* Differences in the hydrophobicity of solutes and polymers drive partitioning. *Steric exclusion:* Larger molecules may preferentially partition into one phase due to steric hindrance. *Affinity and solubility:* Specific affinity of solutes for one of the polymers influences partitioning.	[71,72]
Polymer–salt	*Electrostatic interactions:* Charge interactions between solutes and salt ions can drive partitioning. *Hydration and solubility:* Differences in solute hydration and solubility in the polymer and salt-rich phases. *Salting out effect:* Salts can cause phase separation by reducing solubility of polymers and solutes.	[73,74]
Alcohol–salt	*Hydrophobic and hydrophilic balance:* Partitioning influenced by solute hydrophobicity and alcohol properties. *Electrostatic interactions:* Interaction of solutes with salt ions. *Solvent solubility:* Solubility of solutes in alcohol versus salt-rich aqueous phase.	[75,76]
Ionic liquid–salt	*Electrostatic and ionic Interactions:* Strong electrostatic interactions between solutes and ionic liquids or salts. *Hydrophobic and hydrophilic balance:* Partitioning influenced by solute hydrophobicity and ionic liquid properties. *Complex formation:* Ionic liquids can form complexes with solutes, affecting partitioning.	[69,77,78]
Ionic liquid–polymer	*Combination of polymer and ionic liquid interactions:* Both hydrophobic and electrostatic interactions play roles. *Hydrogen bonding:* Interaction of solutes with hydrogen bonding sites in polymers and ionic liquids. *Solubility and affinity:* Specific solubility and affinity of solutes for either phase.	[69,77,78,79,80,81]

**Table 4:** Key variables affecting the enzyme separation and maintaining enzyme activity in ATPS and strategies for their optimization.

Key Variables	Impact	Optimization Strategies	Reference
pH	The pH affects the charge and solubility of both the enzyme and the phase-forming polymers or salts.	Determine the isoelectric point (pI) of the enzyme and adjust the pH to enhance partitioning into the desired phase. Maintain a pH that ensures the enzyme remains in its active and stable form. Perform pH titrations to find the optimal range for maximal partitioning and stability.	[90,91,92]
Temperature	Temperature influences enzyme activity, stability, and the phase behavior of the ATPS.	Identify the temperature range where the enzyme is most stable and active. Adjust the temperature to balance enzyme stability with the thermodynamics of phase separation.	[91,92,93,94,95]
Concentration of phase-forming components	The concentration of phase-forming components determines the phase separation characteristics and the volume ratio of the phases.	Vary the concentrations of the polymers or salts to achieve a sharp phase separation with a high partition coefficient for the enzyme. Optimize the concentration to minimize enzyme denaturation or inactivation. Use response surface methodology (RSM) to systematically vary concentrations and analyze the effects on enzyme partitioning and stability.	[91,92,95,96]
Ionic strength	Ionic strength can affect enzyme solubility, charge interactions, and phase behavior	Adjust the ionic strength to enhance enzyme partitioning into the preferred phase without compromising stability. Use salts that promote phase separation and increase enzyme stability. Conduct experiments to determine the optimal ionic strength for the specific enzyme and ATPS used.	[49,97]
Additives and co-solvents	Additives such as stabilizers, detergents, or co-solvents can enhance enzyme stability and influence partitioning behavior.	Identify additives that protect the enzyme from denaturation and enhance partitioning. Optimize the concentration of additives to achieve the desired balance between stability and partitioning efficiency. Test different additives and co-solvents in small-scale experiments to identify the most effective ones.	[95,96]

**Table 5:** Application of ATPSs for enzymes/proteins separation and purification.

ATPS	Enzyme/Protein	Source	Extraction Efficiency	Reference
polyethylene glycol (PEG 4000, 6000, and 10,000) and ammonium sulfate (6.60%, 7.26%, 7.92%, and 8.26%)	lipase	*Bacillus* strain isolated from soil	Optimal extraction conditions to ensure maximum efficiency were 12.5% PEG 10,000 and 7.92% ammonium sulfate. In the top phase (rich in PEG), 78.3% of the lipase was recovered.	[101]
ATPS based on polyethylene glycol and citrate buffer, with ionic liquids (ILs) as adjuvants	L-asparaginase	*Escherichia coli*	5% of 1-butyl-3-methylimidazolium methanesulfonate [C_4_mim][CH_3_SO_3_] as adjuvant lead to 87.94 ± 0.03% recovery and specific activity of 3.61 ± 0.38 U/mg protein	[102]
ATPS based on tetrabutylammonium bromide, tetrabutylammonium chloride, choline chloride, and betaine + potassium phosphate buffer	lipase and protease	fermentation broth of *Yarrowia lipolytica*	Extraction efficiencies of 100% for lipase and 96.87% for protease were achieved in a single step using tetrabutylammonium chloride based ATPS.	[62]
ATPS based on polyethylene glycol and potassium phosphate functionalized with cysteine	laccase	model solution	88% extraction efficiency	[103]
ATPS containing 16% (*w*/*w*) PEG2000 and 15% (*w*/*w*) (NH_4_)_2_SO_4_ at pH 6.0	prolyl endopeptidase	fermentation broth	Enzyme recovery of 79.74% and purification coefficient of 7.64	[104]
PEG (4000 and 6000) + K_2_HPO_4_/H_3_PO_4_ + water and 2-propanol + K_2_HPO_4_/H_3_PO_4_ + water in pH 7.0	lipase	porcine pancreas	The best ATPS for porcine pancreatic lipase partition was composed of 13% of PEG 4000 and 9% of K_2_HPO_4_/H_3_PO_4_. Described system ensured enzymatic activity of 0.056, theoretical recovery index of 94.655% and purification factor of 4.357.	[105]
ammonium sulfate, sodium citrate, sodium sulfate, and magnesium sulfate (10%, 15%, 20%, and 25% *w*/*v*) + 15% (*w*/*w*) PEG 4000	lipase	Asian seabass liver	20% ammonium sulfate (*w*/*v*) and 50% PEG-6000 (*w*/*w*) ensured 48% lipase yield	[106]
PEG 1500 or 4000 and phosphate	protease	ora-pro-nobis		[107]
PEG 6000 + ammonium sulphate and sodium sulphate	papain enzyme (cysteine protease)	papaya peel	PEG 6000 + 18% (*w*/*w*) Na_2_SO_4_ at pH 9 ensured extraction yield of 26.38%	[108]
alcohol-/salt-based aqueous two-phase system	lipase	*Bacillus cereus* strains isolated from *Tagetes minuta* root soil	ATPS with xylitol (45% (*w*/*w*)) and potassium phosphate (90% (*w*/*w*)) ensured the purification factor of 24.14 and yield of 87.71%	[109]
PEG (1500 and 6000) + ammonium sulfate, sodium sulfate, dipotassium hydrogen phosphate, and disodium hydrogen phosphate	peroxidase	cabbage	The partition coefficient of the enzyme 15.75 ± 4.07, the percent yield of 97.06 ± 0.71, and the purification factor of 4.86 ± 0.70 times were obtained using of 16% (*w*/*v*) ammonium sulfate salt and 25% (*w*/*v*) of PEG 1500	[110]
pyrrolidinium formate and propionate + K_3_PO_4_ and K_2_HPO_4_	hemoglobin, cytochrome C, α-chymotrypsin and albumin	model solution	ATPS composed of pyrrolidinium formate and K_2_HPO_4_ was selected for the protein extraction and distribution rate of proteins was the highest in hemoglobin	[111]
PEG (400, 600, 1000, and 2000) or polypropylene glycol (400) or the copolymers (Pluronic PE6200, PE6400 Pluronic L35,UCON (PEG-ran-PPG)+ citrate buffered salt	pentraxin-3 holds	human serum	ABS-TPP formed by PEG 1000 and K_3_C_6_H_5_O_7_/C_6_H_8_O_7_ allows to simultaneously deplete high abundance serum proteins and completely extract PTX-3 in the polymer-rich top phase	[112]
low cytotoxic butylguanidinium chloride ionic liquid and different salts (KH_2_PO_4_, K_2_HPO_4_, K_3_PO_4_)	biomarkers in saliva	human saliva	butylguanidinium chloride and K_2_HPO_4_ ATPS ensured extraction efficiencies higher than 80.5% and reduced the limits of detection down to 0.40 ng/mL	[113]
PEG 4000 + sodium citrate, and polyethylene glycol 8000 + sodium phosphate	bovine serum albumin	model solution	the maximum recovery percentage and partition coefficient were 98.99% and 97.69, using PEG4000 concentration 1.5 g/10 mL and sodium citrate concentration 2.7 g/10 mL	[114]
poly(ethylene glycol-ran-propylene glycol) monobutyl ether (EOPO) + magnesium sulfate, sodium citrate, or potassium phosphate	lipase	Nile tilapia (*Oreochromis niloticus*)	20% crude enzyme, 40% EOPO 3900, 10% (NH_4_)_2_SO_4_ and 4% NaCl system ensured the total yield of 93.59% at pH 8.5 and 40 °C	[115]
Alcohol (ethanol, 2-propanol and, 1-propanol) + salts (sulphate, phosphate, and citrate)	xylanase	*Bacillus subtilis*	Highest coefficient of partition of 6.58 and selectivity of 4.84 were obtained of ATPS composed of 26% (*w*/*w*) 1-propanol, 18% (*w*/*w*) ammonium sulphate.	[116]
PEG/trisodium citrate dehydrate (NaCit) ATPS	xylanase	*Bacillus subtilis* fermentation broth	The 96% of xylanase recovery in the PEG phase with the maximum purification factor of 2.17 and partition coefficient of 69.87 were obtained	[117]
ATPSs composed of (i) potassium phosphate and ethanol and (ii) sodium citrate and ethanol	total proteins	*Arthrospira platensis*	ATPS including 19% potassium phosphate and 30% ethanol resulted in 1.27 and 77.45% recovery, while ATPS based on 19.5% sodium citrate and 29% ethanol resulted in 1.31 purity and 78% recovery	[118]
PEG and potassium phosphate and sodium citrate, alcohol (ethanol, n-propanoland isopropanol), and salt (ammonium sulfate, potassium phosphate, and sodium citrate)	pectinases (exo-polygalacturonase, pectinmethylesterase, and pectin lyase)	*Aspergilus niger* ATCC 9642	The crucial parameters for the purification of pectinases, namely pH, PEG molecular weight, and salt content, determine the effectiveness of aqueous two-phase systems.	[119]

**Table 6:** Application of ATS systems for biotransformations.

ATPS	Reaction	Efficiency	Reference
10 % (*w*/*v***)** PEG 3000 + 15 *w*/*v* % dextran	Enzymatic oxidation of uric acid by urate-oxidase, which produced peroxide that was subsequently converted in a horseradish peroxidase-mediated oxidation of guaiacol and ABTS.	strong influence of the substrate–polymer interactions on the diffusion rates and enzyme kinetics	[154]
sodium polyacrylate (NaPA), ethylene oxide/propylene oxide (EO/PO) polymers, and (EO)*_x_*-(PO)*_y_*-(EO)*_x_* triblock copolymers composed ATPS	Enzymatic (levansucrase) production of fructooligosaccharide	fructooligosaccharide was purified with high yields (72.94–100%) depending on different triblock copolymers	[155]
ionic liquid cholinium dihydrogen phosphate and the polymer PEG 600	Laccase-catalyzed rutine oligomerisation	rutin oligomerization yields: 95% in the first cycle, 91% in the second cycle, and 89% in the last cycle	[156]
ATPS including 1-butyl-3-methylimidazolium ionic liquids with five kinds of different anions (Cl^−^, H_2_PO_4_^−^, Br^−^, BF4^−^, and HSO_4_^−^) + buffer	Biotransformation from pieced to resveratrol using immobilized edible *A. oryzae* cells	the conversion rate of pieced reached 85.21%	[157]
1-allyl-3-methylimidazolium chloride + NaH_2_PO_4_/Na_2_HPO_4_	Enzymatic saccharification of crystalline cellulose	the final yield of glucose was about 70%	[158]
20% ethylene oxide/80% propylene oxide (*v*/*v*) random copolymer (EO_20_PO_80_) ATPS	Enzymatic synthesis of cefprozil	the yield of the enzyme reaction was 93.1%	[159]
*n*-hexadecane-ATPS	Enzymatic production(*R*)-mandelic acid from styrene biobased *L*-phenylalanine, glycerol, and glucose	(*R*)-mandelic acid at 1.52 g/L was produced from styrene in >99% enantiomeric excess	[160]

**Table 7:** Main challenges and proposed solutions when working with ATPS.

Common Challenge	Challenge Description	Proposed Solution	Reference
Enzyme stability	*Thermal stability:* ATPS can be sensitive to temperature variations, which may lead to denaturation or loss of activity. *pH sensitivity:* The activity of ATPS is highly dependent on the pH of the environment. *Oxidative damage:* ATPS can be prone to oxidative damage, which affects its functionality.	*Protein engineering:* Modifying the enzyme through site-directed mutagenesis to enhance thermal and pH stability. *Chemical stabilizers:* Using additives that protect ATPS from denaturation and oxidative damage. *Immobilization:* Attaching ATPS to solid supports to improve stability and reusability.	[97,161,162,163]
Process optimization	*Substrate availability:* Ensuring a continuous and optimal supply of substrates (ADP and inorganic phosphate) is crucial. *Product inhibition:* Accumulation of ATP can inhibit ATPS activity, creating a feedback loop that reduces efficiency. *Membrane integrity:* Maintaining the integrity of the lipid bilayer in membrane-bound ATPS systems is critical for their function. *Energy efficiency:* Efficient conversion of energy sources (e.g., proton gradient) to ATP is necessary for the system’s overall effectiveness.	*Optimizing conditions:* Fine-tuning pH, temperature, and substrate concentrations to maximize ATPS activity. *Feedback control systems****:*** Implementing systems that monitor ATP levels and adjust inputs to prevent product inhibition. *Synthetic membranes****:*** Developing robust synthetic membranes that can withstand industrial conditions while maintaining ATPS functionality. *Co-factor recycling****:*** Using co-factor regeneration systems to maintain a consistent supply of substrates and remove inhibitory products.	[96,97,164,165]
Scalability	*Economic viability:* Scaling up ATPS systems for industrial applications can be cost-prohibitive. *System integration:* Integrating ATPS into larger biotechnological processes can be complex and require precise control.	*Cost-effective production****:*** Developing methods for the economical production and purification of ATPS. *Modular system design****:*** Creating modular ATPS units that can be easily integrated and scaled within various biotechnological processes. *Process automation****:*** Implementing advanced control systems to automate and optimize the operation of ATPS systems at scale.	[166]

**Table 8:** Examples of successful industrial applications of ATPS.

Used ATPS	Process	Process Efficiency	Reference
Detergent (Agrimul NRE 1205) and (NH_4_)H_2_PO_4_ based ATPS	Separation of the proteins (EGIcore-HFBI and the small protein hydrophobin I, expressed in *Trichoderma reesei*) from culture broth on the 1200 L scale	The partition coefficient and the concentration factor were equal in the 10 mL and 1200 L scale separation. Used ATPS ensured recovery of 62% and a purification factor of 3.5.	[174]
PEG–potassium phosphate ATPS	Recovery of B-phycoerythrin, a natural high-value pigment from *Porphyridium cruentum* on pilot plant scale of 8.55 kg.	29% (*w*/*w*) PEG 1000 g/mol and 9% (*w/w*) potassium phosphate based ATPS rendered a recovery yield of 84% and a 2.3 purification fold	[175]
1-propanol-ammonium sulphate-based ATPS	Recovering lipase from the fermentation broth of *Burkholderia cepacia* on pilot plant scale of 5 L	Purification factor of 12.2, a separation efficiency of 93% and a selectivity of 40	[176]
polyethylene glycol 3350-potassium phosphate ATPS	Recovery of human immunoglobulin G in continuously operated mixer-settler device	Average IgG recovery of 65 ± 17%. Continuous operation processed 1 kg of ATPS in a 12 min run	[177]

## Data Availability

Data will be available on request.
